# UPLC-MS/MS method for simultaneous determination of pyrantel, praziquantel, febantel, fenbendazole and oxfendazole in dog plasma and its application to a bioequivalence study

**DOI:** 10.3389/fphar.2025.1544215

**Published:** 2025-02-13

**Authors:** Fengyichi Zhang, Fanxi Guo, Xinyu Zhao, Chongyang Li, Junli Wang, Mengyun Wang, Ruixia Ma, Baochang Chen, Qiangqiang Miao, Yimeng Wang, Zihan Wang, Di Cao, Zugong Yu

**Affiliations:** Laboratory of Veterinary Pharmacology and Toxicology, Department of Basic Veterinary Medicine, College of Veterinary Medicine, Nanjing Agricultural University, Nanjing, China

**Keywords:** pyrantel, praziquantel, febantel, fenbendazole, oxfendazole, UPLC-MS/MS, bioequivalence, dog plasma concentration

## Abstract

A rapid, sensitive and reliable ultra–performance liquid chromatography–tandem mass spectrometry (UPLC–MS/MS) was developed and validated for the simultaneous quantitation of pyrantel (PYR), praziquantel (PRA), febantel (FBT) and its active metabolites, fenbendazole (FEN) and oxfendazole (OXF) in dog plasma. Fenbendazole–D3 (FEN–D3) was used as an internal standard (IS). The analytes and IS were prepared using simple protein precipitation (PP) combined with liquid–liquid extraction (LLE). Chromatographic analysis was performed on UPLC BEH C_18_ column using acetonitrile–0.1% formic acid in water for gradient elution. Detection was carried out in multiple reaction monitoring (MRM) mode under positive electrospray ionization. The standard curves were linear through the concentration range of 4–240 ng/mL for PYR and OXF, 15–900 ng/mL for PRA, 2–120 ng/mL for FBT and 10–600 ng/mL for FEN with all correlation coefficients >0.99. The intra–and inter–batch precision was 1.08%–14.26% and accuracy was from 90.66% to 110.28%. The mean extraction recoveries for the analytes and IS were >90%. The total run time was 9.0 min. The developed method was successfully applied to a bioequivalence study after oral administration of compound febantel tablets in 38 healthy dogs.

## 1 Introduction

Intestinal parasitic mixed infections are common in dogs. Regular deworming has become an established routine in the care regimen of companion animals. For mixed infection, the effect of a single anthelmintic drug is limited, and the combination of anthelmintic drugs is more effective in controlling mixed anthelmintic infection. A classic drug combination consisting of pyrantel pamoate, PRA, and FBT has been widely used in routine deworming of dogs for more than 20 years, which can treat all relevant helminths of dogs simultaneously (Barr et al., 1998).

Compound febantel tablets are broad–spectrum anthelmintic drugs, which are composed of three medications: pyrantel pamoate, PRA and FBT. It was approved by the U.S. Food and Drug Administration in 1994 for removing intestinal parasites such as tapeworms, hookworms and ascarids in dogs (U.S. Food and Drug Administration, 1996). Besides, the European Medicines Agency has approved the compound febantel tablets produced by Chanelle Pharmaceuticals Manufacturing Ltd. for the treatment of various parasitic infections in dogs caused by nematodes and cestodes (Europrean Medicines Agency, 2012).

**FIGURE 1:**
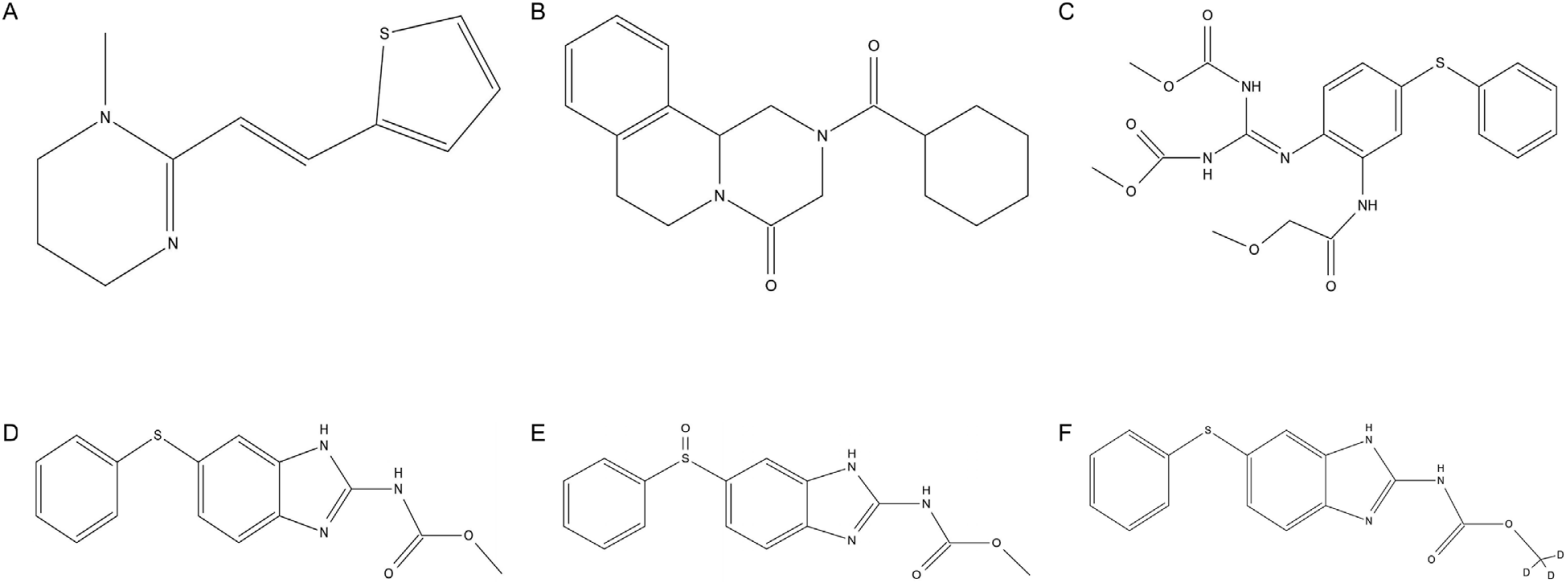
Chemical structures of **(A)** pyrantel, **(B)** praziquantel, **(C)** febantel, **(D)** fenbendazole, **(E)** oxfendazole and **(F)** fenbendazole–D3.

PYR (Figure 1A) is a tetrahydropyrimidine nicotinic agonist anthelmintic to treat intestinal infections caused by hookworms and roundworms (Kopp et al., 2008). Pyrantel pamoate is the salt of the tetrahydropyrimidine base and pamoic acid which has low systemic absorption due to its poor absorption from the gastrointestinal tract (Pitts and Migliardi, 1974; Gokbulut et al., 2014). It causes sustained spastic contraction and paralysis of worms by depolarizing the myoneural junction and inhibiting cholinesterase (Prichard and Ranjan, 1993). PRA (Figure 1B) exhibits broad spectrum activities against parasitic trematodes and cestodes (Cioli and Pica-Mattoccia, 2003). The molecular mechanism of action of PRA remains unclear. Recent research results indicate that a flatworm transient receptor potential ion channel from the melastatin subfamily has been identified as a target for the action of PRA (Marchant, 2024). FBT (Figure 1C), a pro–benzimidazole anthelmintic, has been widely used for treatment of gastrointestinal infections caused by nematodes and cestodes in animals (Beretta et al., 1987). The anthelminthic activity of FBT is due to its *in vivo* metabolism to FEN (Figure 1D) and OXF (Figure 1E) (Landuyt et al., 1993). As it is, FBT also possesses an anthelmintic effect due to its direct neurotoxic action (DiPietro and Todd, 1987).

Currently, a new compound febantel tablet formulation, with each tablet containing 150 mg FBT, 144 mg pyrantel pamoate and 50 mg PRA has been developed to treat canine intestinal parasite infections. In order to demonstrate the clinical efficacy of this new formulation, a bioequivalence study was carried out in this study, with Drontal^®^ Plus Tasty developed by BAYER as the reference formulation. So for monitoring the pharmacokinetics of the test formulation in clinical trials and bioequivalence study, it was necessary to develop a simple, rapid, specific, selective and sensitive analytical method for the quantification of PYR, PRA, FBT, FEN and OXF in dog plasma.

Bibliographic data on the simultaneous determination of two or more of the aforementioned substances in animal plasma are highly limited. Landuyt et al. (1993) developed a liquid chromatography–ultraviolet detection (HPLC–UV) method for the determination of FBT and its major metabolites in lamb plasma with lower limit of quantification (LLOQ) 50 ng/mL for FBT and 5 ng/mL for the metabolites which used a LLE procedure. Gokbulut et al. (2007) reported a HPLC–UV method for the quantification of FEN, OXF and albendazole in dog plasma with LLOQ 50 ng/mL which also involved a LLE procedure. Morovján et al. (1998) reported a solid–phase extraction (SPE) HPLC–UV method for the determination of FEN, PRA and PYR in dog plasma with limits of quantitation in the range of 15–25 ng/mL. Arion et al. (2018) applied a liquid chromatography–mass spectrometry–high–throughpuy screening (UPLC–MS–HTS) method for the quantification of PYR and PRA in a pharmacokinetic study in cat plasma demonstrating LLOQ 6 ng/mL for PYR and 20 ng/mL for PRA.

To the best of our knowledge, there is only one HPLC–MS method for the simultaneous determination of PYR, PRA, FBT, FEN and OXF in dog plasma. SPE was used for sample preparation with the average recoveries of 77%–94% in the above–mentioned study (Klausz et al., 2015). However, this extraction method is not only time–consuming but also costly. Moreover, the detection of a single sample takes up to 23 min. These factors will all cause inconvenience to clinical application. UPLC–MS/MS is more sensitive than HPLC–MS in terms of the analytical method and has obvious advantages in pharmacokinetic studies. Its powerful separation and analysis capabilities are suitable for *in vivo* metabolism of complex drug systems. There are no UPLC–MS/MS methods for the determination of these analytes in dog plasma. Hence, we describe development and validation of a simple, rapid and sensitive method for simultaneous quantification of PYR, PRA, FBT, FEN and OXF using a deuterated internal standard (FEN–D3, Figure 1F). The method presents an efficient plasma extraction procedure based on LLE and PP techniques and has a shorter analysis run time (9 min) and a very low plasma volume (0.1 mL). The proposed method has been successfully applied to a bioequivalence study of compound febantel tablet formulation in 38 beagle dogs under fasting condition.

## 2 Materials and methods

### 2.1 Chemicals and reagents

Pyrantel pamoate (purity 99.7%) standard product was purchased from National Institutes for Food and Drug Control (Beijing, China). Standard products of PRA (purity 99.6%), FBT (purity 99.7%) and FEN (purity 100%) were purchased from China Institute of Veterinary Drug Control (Beijing, China). OXF (purity 96.75%) standard product was purchased from Dr. Ehrenstorfer GmbH (Augsburg, Germany). FEN–D3 standard product, as an internal standard (IS), was purchased from Shanghai Zhenzhun Biomedical Technology Co., Ltd. (Shanghai, China). HPLC grade acetonitrile and methanol were obtained from Merck (Darmstadt, Germany). LC–MS grade formic acid was obtained from Anaqua Chemicals Supply (Wilmington, DE, United States). *N*, *N*–dimethyl–formamide (DMF) was procured from Macklin (Shanghai, China). LC–MS grade ammonia solution was procured from Aladdin (Shanghai, China). Analytical grade ethyl acetate was procured from Guangdong guanghua Sci–Tech Co., Ltd. (Guangdong, China). Deionized water used during the entire analysis was purified using a Direct–Q5 UV system from Millipore (Boston, MA, United States). Blank dog plasma used in the experiment was collected from different beagle dogs and stored at −80°C until use (The Experimental Animal Center of Nanjing Agricultural University, Nanjing, China).

### 2.2 Instruments and UPLC–MS/MS conditons

Quantitative analysis was performed on an ACQUITY™ UPLC system (Waters Corp., Milford, MA, United States), coupled with a Waters Xevo TQD triple quadrupole mass spectrometer (Waters Corp., Milford, MA, United States) equipped with electrospray ionization (ESI) and a Nitrogen generator (Peak Corp., Glasgow, United Kingdom). The analytes were separated on Waters Acquity UPLC BEH C_18_ (2.1 × 50 mm, 1.7 μm) with a thermostated column oven maintained at 35°C. The mobile phase consisted of solvent A (acetonitrile) and solvent B (0.1% formic acid) at a flow rate of 0.4 mL/min according to the following linear gradient: 0–6.0 min, 10%–100% A; 6.0–6.5 min, 100% A; 6.5–7.0 min, 100%–10% A; 7.0–9.0 min 10% A. The autosampler temperature was set at 6°C, and the injection volume was 5 μL.

Mass spectrum analysis was performed in the positive ionization mode under multiple reaction monitoring by monitoring ion transitions of *m/z* 207.1 > 150 for PYR, *m/z* 313.2 > 203.1 for PRA, *m/z* 447.1 > 415.1 for FBT, *m/z* 300 > 268.1 for FEN, *m/z* 316 > 159 for OXF and *m/z* 303 > 268.1 for IS, respectively. The source parameters for mass spectrum analysis were as follows: capillary voltage, 1.0 kV; source temperature, 150°C; desolvation temperature, 500°C; desolvation gas flow, 800 L/h; cone gas flow, 50 L/h; dwell time, 80 ms. The cone voltages were 40 V for PYR, FEN, OXF and IS, 35 V for PRA and 30 V for FBT, and collision energies were 27 eV for PYR, 15 eV for PRA, 13 eV for FBT, 20 eV for FEN, 36 eV for OXF and 20 eV for IS, respectively. Mass Lynx software version 4.1 was used to controll all UPLC and MS parameters.

### 2.3 Preparation of standard stock, calibration standards and quality control samples

Individual stock solutions of PYR, PRA, FBT, FEN and OXF were prepared for standards and quality control samples in DMF at 1 mg/mL. IS stock solution was also prepared in DMF at 400 μg/mL. The mixed working solutions were obtained by mixing the stock solution in different volumes and then performing appropriate dilution in methanol: water (50:50, *V*/*V*). The IS working solution of 200 ng/mL was also prepared with DMF from the primary stock solution. All the primary stock solutions were stored at −20°C for more than 2 months. The working solutions for the analytes and IS were stored at 4°C and brought to room temperature before use.

Calibration standards (CSs) and quality control (QC) samples were prepared by spiking 10 μL mixed woking solutions to 90 μL of blank dog plasma. CSs of PYR and OXF were made at 4, 8, 16, 40, 80, 160 and 240 ng/mL concentrations, while QC samples were prepared at 180 ng/mL (high quality control, HQC), 120 ng/mL (middle quality control, MQC), 12 ng/mL (low quality control, LQC) and 4 ng/mL (LLOQ). For PRA, CSs were made at 15, 30, 60, 150, 300, 600 and 900 ng/mL concentrations, while QC samples were prepared at 675 ng/mL (HQC), 450 ng/mL (MQC), 45 ng/mL (LQC) and 15 ng/mL (LLOQ). For FBT, CSs were made at 2, 4, 8, 20, 40, 80 and 120 ng/mL concentrations, while QC samples were prepared at 90 ng/mL (HQC), 60 ng/mL (MQC), 6 ng/mL (LQC) and 2 ng/mL (LLOQ). For FEN, CSs were made at 10, 20, 40, 100, 200, 400 and 600 ng/mL concentrations, while QC samples were prepared at 450 ng/mL (HQC), 300 ng/mL (MQC), 30 ng/mL (LQC) and 10 ng/mL (LLOQ).

### 2.4 Internal standard selection

FEN–D3 was selected as an internal standard. FEN–D3 is highly similar in chemical structure to FEN. When there are complex matrix components in the sample, FEN-D3 can undergo similar physicochemical processes together with FEN, reducing the errors caused by the matrix effect. For PYR, PRA, FBT and OXF, although their chemical structures are not exactly the same as that of FEN, the stability and behavioral characteristics of FEN-D3 in the entire detection system can serve as a reference standard. During the simultaneous detection of multiple components, the stability of its chemical properties helps to provide a relatively stable signal in the complex instrumental analysis environment, which can be used to compare and calibrate the detection signals of other drugs.

### 2.5 Sample preparation

For analysis, 100 μL of plasma was mixed with 10 μL of IS woking solution (200 ng/mL), followed by vortexing for 10 s. Then, 50 μL of 1 M ammonia solution and 50 μL of DMF were added and vortexed for 30 s. After vortexing, 500 μL of acetonitrile and 800 μL of ethyl acetate were added, then vortexed for 5 min, and centrifuged at 12,000 r/min for 7 min at 4°C. The supernatant was transferred to a new 5 mL centrifuge tube. In the remaining precipitation, 200 μL of acetonitrile and 800 μL of ethyl acetate were added, then vortexed for 5 min and centrifuged at 12,000 r/min for 10 min at 4°C. The two supernatants were mixed in the 5 mL centrifuge tube and then evaporated to dryness under nitrogen stream at 40°C. The residue was reconstituted with two dilution solvents containing 500 μL of acetonitrile: water (1:1, *V*/*V*) and 500 μL of acetonitrile: water (2:8, *V*/*V*). After samples were filtered by 0.22 um filter membrane, a 5 μL aliquot was injected into the UPLC–MS/MS system.

### 2.6 Method validation

Validation of the quantitative UPLC–MS/MS method was assessed including specificity and selectivity, linearity, precision and accuracy, recovery, matrix effect, dilution reliability and stability according to U.S. Food and Drug Administration Guidance for Industry (U.S. Food and Drug Administration, 2018), and National Medical Products Administration Technical Guideline for Non–clinical Pharmacokinetic Study of Chemical Drugs (National Medical Products Adminstration, 2005).

#### 2.6.1 Specificity and selectivity

The specificity and selectivity of this method was documented by analyzing six blank plasma samples from different dogs and spiked plasma samples at the LLOQ concentrations and the upper limit of quantification (ULOQ) concentrations for each analyte. The peak area of the interference components should be less than 20% of the peak area of the LLOQ standard and less than 5% of the peak area of the IS.

#### 2.6.2 Linearity

Calibration curves were acquired by plotting the peak area ratios of the transition pair of analytes to that of IS versus the nominal concentration using a linearly weighed (1/*x*) least squares regression method in duplicate. Calibration curves were considered acceptable when the correlation coefficient (*r* ^2^) was greater than 0.99 and the acceptance criterion for each back–calculated standard concentration was ±15% deviation from the nominal value except at LLOQ, which was set at ± 20%.

#### 2.6.3 Precision and accuracy

Intra–batch and inter–batch precision and accuracy were determined by analyzing five replicates at LLOQ in addition to three different QC levels as described above on three consecutive days. The mean accuracy should be within ±15%, except for the LLOQ where it can be within ±20% of the nominal concentration. Similarly, the precision (coefficient of variation, CV) should not exceed 15%, except for the LLOQ where it can be less than 20%.

#### 2.6.4 Recovery

The extraction recoveries of PYR, PRA, FBT, FEN, OXF and IS from dog plasma were evaluated in six replicates by comparing the mean peak area responses of pre–extraction fortified samples at LQC, MQC and HQC levels with those of post–extraction fortified samples at the same concentrations.

#### 2.6.5 Matrix effect

The matrix factors of each analyte and IS were determined by comparing the mean peak area responses of post–extraction fortified samples at three QC levels with those of solutions prepared in moblile phase solutions (neat samples) at equivalent concentrations. The IS–normalized matrix factors were calculated by dividing the matrix factor of the analytes by the matrix factor of the IS. Since it is impossible to completely eliminate the effect of the matrix, the consistency in matrix effect is examined in different plasma sources. The precision should be ≤15% for the IS–normalized matrix factors at each level.

#### 2.6.6 Dilution reliability

Dilution reliability was investigated to ensure that samples with concentrations above ULOQ could be diluted with blank matrix without affecting accuracy and precision. PYR and OXF were spiked at a concentration of 720 ng/mL in dog plasma, while PRA, FBT and FEN were spiked at concentrations of 2,700, 360 and 1,800 ng/mL. All of the analytes were diluted with pooled dog plasma five folds in five replicates and analyzed. Acceptance criteria were defined as precision (CV%) not exceeding 15%, and accuracy being within ±15% of the nominal concentration.

#### 2.6.7 Stability experiments

Stability experiments were conducted in working solutions of the analytes and IS for short term stability at 4°C and stock solutions for long term stability at −20°C, respectively. Peak area ratio responses (Analytes/IS) of stored solutions were compared with those of freshly prepared solutions. The acceptance criterion was ±10.0% deviation when comparing the values of fresh solutions. The stabilities of the analytes in beagle dog plasma were evaluated by analyzing five replicates at LQC and HQC levels. Types of stabilities were following as: bench top stability (room temperature), refrigerator stability (4°C), autosampler stability for processed samples (6°C), freeze–thaw stability (−80°C), short term stability (−20°C) and long term (–80°C) storage stability. The samples were considered stable if the accuracy was within ±15% of the nominal concentration and the precision (CV%) were ≤15%.

#### 2.6.8 Carry–over

Carry–over effect was estimated by injecting blank samples after injecting calibration standards at the ULOQ concentrations. The measured peak area should not exceed 20% of the LLOQ and 5% of the IS.

### 2.7 Bioequivalence study and statistical analysis

The study design was an open label, balanced, randomized, crossover, two–treatment, two–period, two–sequence and single–dose study to investigate the bioequivalence between a single oral dose of Drontal^®^ Plus Tasty containing 150 mg FBT, 144 mg pyrantel pamoate and 50 mg PRA (BAYER, Germany) and self–developed compound febantel tablets of the same specification in 40 beagle dogs under fasting conditions. Adaptive feeding and observation were carried out for 7 days before the bioequivalence study. Physical examination, blood routine and blood biochemical tests were performed the day before the trial to ensure the health of the dogs. The study protocol was conducted in accordance with the Ethical Guidelines for Investigations in Laboratory Animals and approved by the Ethics Committee of Nanjing Agricultural University (Nanjing, China). Prior to the study, the dogs were fast and maintained with water for 12 h. The subjects were orally administered a single dose of test or reference formuations with 100 mL of water with a washout period of 2 weeks. Blood samples were collected into heparinized tubes at 0.00 (pre–dose), 0.17, 0.33, 0.67, 1.00, 1.50, 2.00, 2.50, 3.00, 4.00, 5.00, 6.00, 7.00, 8.00, 9.00, 10.00, 11.00, 12.00, 14.00, 24.00 and 30.00 h after oral administration. However, two dogs vomited out pills during blood sample collection in this study. Therefore, only 38 dogs completed the crossover process. The number of blood collections for drug analysis was 21 samples in each study period. The collected blood samples were centrifuged in a pre–cooled centrifuge at 2,150 g for 10 min, and the separated plasma was transferred to a clean centrifuge tube and frozen in a −80°C refrigerator until analysis. During the entire study, subjects had a standard diet while water intake was unmonitored.

The pharmacokinetic parameters for the analytes were evaluated by non–compartmental analysis using Phoenix WinNonlin software version 8.1 (Certara Corporation, Radnor, PA, United States). The maximum plasma concentration (C_max_), the area under the plasma concentration-time curve from time zero to the last sampling time (AUC_0–30_), the area under the plasma concentration-time curve from time zero to infinity (AUC_0–inf_), as the primary target, and their geometric mean ratios (test/reference) using log transformed data were assessed to determine whether the formulations were pharmacokinetically equivalent. The drug formulations were considered pharmacokinetically equivalent if the geometric mean ratios and 90% confidence intervals for these parameters were within 80%–125%.

## 3 Results and discussion

### 3.1 UPLC–MS/MS method development

The aim of this study was to provide a bio–sample detection method for the bioequivalence study of compound febantel tablets in dogs. FEN–D3, as an isotope–labeled internal standard, can maximize the elimination of errors in the analysis process. Considering the following points: (1) FBT is metabolized rapidly and the absorption of PYR is limited, resulting in relatively low blood drug concentrations of both. (2) Given that a substantial quantity of samples is involved in the bioequivalence study, it is essential to minimize the time required for sample pretreatment and elution analysis to the greatest extent possible. (3) As five target analytes need to be determined concurrently, a high level of separation is demanded. Therefore, in order to develop and validate a rapid, sensitive, selective and simple analytical method for the extraction and quantification of PYR, PRA, FBT and its active metabolites, FEN and OXF in dog plasma, the extraction procedure, mass spectrum paramaters and chromatographic separation condition were suitably optimized.

#### 3.1.1 Plasma sample pretreatment

Different extraction methods for the analysis of above–mentioned drugs in animal plasma have been reported for many years, such as PP (Gutiérrez et al., 2017; Zhang et al., 2017; Xie et al., 2023), LLE (Landuyt et al., 1993; Gokbulut et al., 2007; Gokbulut et al., 2014), and SPE (Morovján et al., 1998; Klausz et al., 2015). The method of simultaneously extracting these drugs by using solid phase extraction has been reported (Klausz et al., 2015). However, this method has high plasma volume for processing and complex sample processing. It spends a lot of time and cost on sample processing. Therefore, we looked for a simpler sample pretreatment method to improve the sample detection efficiency and reduce the detection cost. As the PP has the advantage of simplicity and speed of analysis, the initial attempts were done with acetonitrile and methanol as protein precipitants. However, this method did not provide cleaner extracts which were apparent from poor recovery at all QC levels for all analytes. Furthermore, LLE was tested using ethyl acetate. Although the recovery of this method was significantly improved, the liquid chromatography system was prone to clogging after the analysis of a large number of samples. On this basis, PP combined with LLE was explored for sample preparation to reduce processsing procedures and save cost. According to the extraction recovery results, acetonitrile and ethyl acetate were adjusted in proportion to obtain the optimal extraction method. Besides, Plasma volume was also reduced in this method. Only 100 μL of plasma was required for each sample. Before sample processing, 50 μL of 1 M ammonia solution and 50 μL DMF were added to the plasma samples in order to adjust the pH value and increase the solubility of the analytes in the organic phase. Re–extraction was also performed after the first extraction to improve the extraction recovery. The mean extraction recoveries of all the analytes at three QC levels and IS were above 90%.

#### 3.1.2 Mass spectrometry

To attain better responses of the analytes and IS, the mass spectrum parameters were optimized in this study. Standard solutions (100 ng/mL) of the analytes and IS were directly infused into the MS using electrospray ionization (ESI) as the ionization source, respectively. The mass spectrometer was tuned in the positive ionization mode to achieve high sensitivity based on a previous study (Klausz et al., 2015). The predominantly protonated precursor ions for PYR, PRA, FBT, FEN, OXF and FEN–D3 were observed at *m*/*z* 207.1, *m*/*z* 313.2, *m*/*z* 447.1, *m/z* 300.0, *m*/*z* 316.0 and *m*/*z* 303.0 in the full scan Q1 mass spectra, respectively. Fragmentation of protonated precursor ions gave the most abundant and consistent product ions at *m*/*z* 150.0, *m/z* 203.1, *m/z* 415.1, *m/z* 268.1, *m/z* 159.0 and *m/z* 268.1 for PYR, PRA, FBT, FEN, OXF and FEN–D3, respectively. Other mass spectrometry–related parameters were also properly optimized to acquire best responses for the analytes. Therefore, ion pairs of *m*/*z* 207.1 > 150 for PYR, *m*/*z* 313.2 > 203.1 for PRA, *m*/*z* 447.1 > 415.1 for FBT, *m*/*z* 300 > 268.1 for FEN, *m*/*z* 316 > 159 for OXF and *m*/*z* 303 > 268.1 for IS were used for quantification of the analytes with a dwell time of 80 ms.

#### 3.1.3 Chromatographic condition

Chromatographic conditions were optimized to obtain sharp peak shape, high response and short run time for the analytes and IS, with the improvement on base–line noise and reduction on solvent consumption. Different mobile phase composition and mobile phase additives were tested for optimal resolution, speed and sensitivity on Waters Acquity UPLC BEH C_18_ (50 mm × 2.1 mm, 1.7 μm) column. Different conbinations of acetonitrile or methanol and additives such as formic acid and ammonium formate in different volume ratios were tried. It was observed that acetonitrile was selected as the organic modifier since it provided shorter run time and sharper peak shape for the anlytes and IS compared to methanol. What’s more, formic acid provided better peak shape and reponse than ammonium formate in the positive mode. Finally, the best chromatographic conditions were acquired using acetonitrile with 0.1% formic acid (10:90, *V*/*V*) as initial mobile phase under a gradient elution mode. The retention time for PYR, PRA, FBT, FEN, OXF and FEN–D3 was 1.41 min, 3.55 min, 4.28 min, 3.29 min, 2.17 min and 3.29 min, respectively. The reproducibility of retention times for the analytes, expressed as CV, was ≤0.67% for 100 injections on the same column.

### 3.2 Method validation

#### 3.2.1 Specificity and selectivity

**FIGURE 2:**
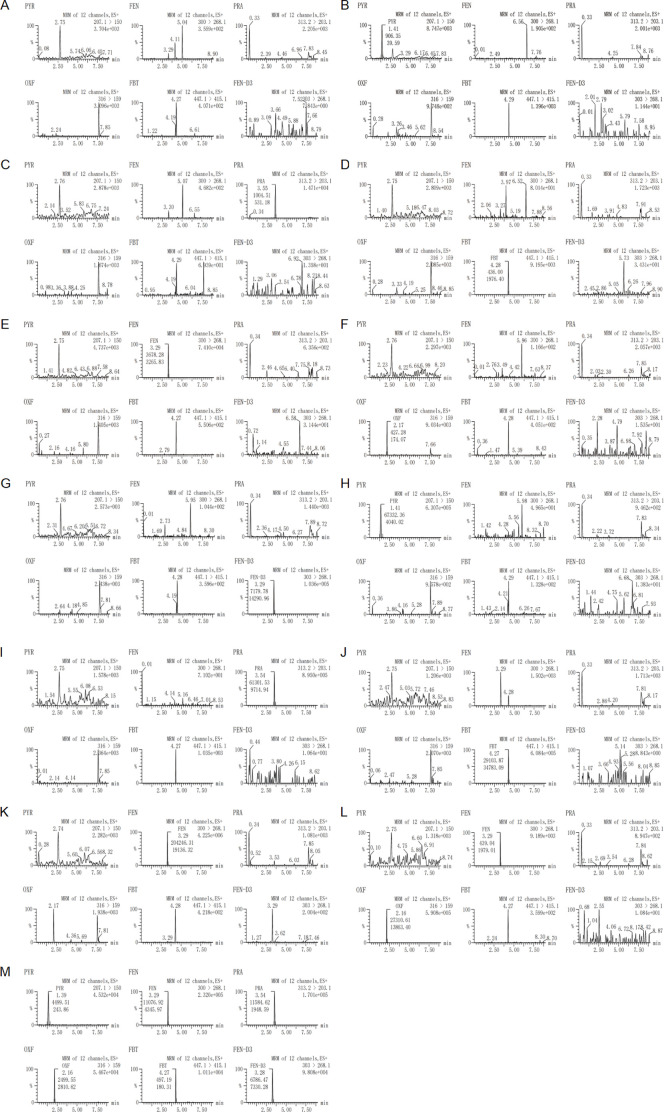
Representative MRM chromatograms of **(A)** a blank plasma sample, **(B)** a plasma sample sipked with pyrantel at the LLOQ, **(C)** a plasma sample sipked with praziquantel at the LLOQ, **(D)** a plasma sample sipked with febantel at the LLOQ, **(E)** a plasma sample sipked with fenbendazole at the LLOQ, **(F)** a plasma sample sipked with oxfendazole at the LLOQ, **(G)** a plasma sample sipked with fenbendazole–D3, **(H)** a plasma sample sipked with pyrantel at the ULOQ, **(I)** a plasma sample sipked with praziquantel at the ULOQ, **(J)** a plasma sample sipked with febantel at the ULOQ, **(K)** a plasma sample sipked with fenbendazole at the ULOQ, **(L)** a plasma sample sipked with oxfendazole at the ULOQ and **(M)** a subject sample after oral administration of a compound febantel tablet formulation (Drontal^®^ Plus Flavor).

The specificity of the method was assessed by the absence of endogenous interfering peaks at retention times of the analytes and the internal standard in 6 different lots of extracted blank plasma. Moreover, The selectivity of the method was demonstrated by the fact that no significant interference peaks of the concomitant drugs or *in vivo* metabolic drugs at the ULOQ levels were found at the retention time of other drugs. Figure 2 shows the typical MRM chromatograms of blank sample, blank plasma spiked with the analytes at the LLOQ, blank plasma spiked with IS, blank plasma spiked with the analytes at the ULOQ and plasma sample from a subject. When detecting the ion pair of OXF, an interference peak appeared at the retention time under the injection of plasma spiked with FEN at the ULOQ. However, The area of the interfering peak was less than 20% of the LLOQ of OXF and had no impact on selectivity.

#### 3.2.2 Linearity and LLOQ

The calibration curve was linear over the range of 4–240 ng/mL for PYR and OXF, 15–900 ng/mL for PRA, 2–120 ng/mL for FBT, 10–600 ng/mL for FEN. A quadratic, 1/x, least–squares regression algorithm was used to plot the peak area ratio (analyte/IS) from MRM versus concentration. The calibration curve equation is *y* = *ax* + *c*, where *y* represents analyte/IS peak area ratio and *x* represents the plasma concentration of the analytes. The representative equations of the calibration curves were *y* = 0.0383196 *x* – 0.0142732, *r* ^2^ = 0.999138 for PYR, *y* = 0.00980901 *x* – 0.00605049, *r* ^2^ = 0.999027 for PRA, *y* = 0.0336798 *x* + 0.00546005, *r* ^2^ = 0.998914 for FBT, *y* = 0.0433366 *x*– 0.0112174, *r* ^2^ = 0.999105 for FEN and *y* = 0.0175517 *x* – 0.00655116, *r* ^2^ = 0.999320 for OXF. The LLOQs were 4 ng/mL for PYR and OXF, 15 ng/mL for PRA, 2 ng/mL for FBT and 10 ng/mL for FEN and were adequate for PK studies following oral administration of compound febantel tablets.

#### 3.2.3 Precision and accuracy

The intra–batch and inter–batch precision and accuracy results for the analytes across four levels are shown in Table 1. The intra–batch precision and accuracy for PYR ranged from 2.33% to 5.17% and 103.89%–110.28%, for PRA ranged from 2.83% to 10.60% and 101.00%–104.88%, for FBT ranged from 2.47% to 14.26% and 90.66%–101.73%, for FEN ranged from 1.87% to 5.56% and 98.70%–100.90%, for OXF ranged from 1.08% to 11.33% and 98.13%–106.94%, respectively. Whereas the inter–batch precision and accuracy for PYR were within 4.03%–8.52% and 99.00%–102.51%, for PRA were within 3.01%–7.63% and 101.73%–103.82%, for FBT were within 4.65%–12.75% and 99.05%–100.79%, for FEN were within 3.06%–5.36% and 99.14%–101.55%, for OXF were within 3.74%–9.12% and 99.66%–104.52%, respectively.

**TABLE 1:** Intra–batch and inter–batch precision and accuracy for the analytes.

Analyte	Level	Concentration added (ng/mL)	Intra–batch (n = 5; single batch)			Inter–batch (n = 15; 5 from each batch)		
			Mean conc. found (ng/mL)	CV (%)	Accuracy (%)	Mean conc. found (ng/mL)	CV (%)	Accuracy (%)
Pyrantel	LLOQ QC	4	4.41	5.17	110.28	4.10	8.52	102.51
	LQC	12	12.52	2.38	104.33	11.88	6.56	99.00
	MQC	120	124.67	2.33	103.89	120.51	5.20	100.42
	HQC	180	189.06	3.36	105.03	182.26	4.03	101.25
Praziquantel	LLOQ QC	15	15.43	10.60	102.86	15.57	7.63	103.82
	LQC	45	45.45	3.38	101.00	45.78	3.01	101.73
	MQC	450	466.89	2.83	103.75	458.34	4.31	101.85
	HQC	675	707.93	3.39	104.88	690.81	3.03	102.34
Febantel	LLOQ QC	2	1.81	14.26	90.66	2.00	12.75	100.08
	LQC	6	5.94	7.72	99.07	6.03	8.26	100.51
	MQC	60	59.90	3.71	99.83	59.43	4.65	99.05
	HQC	90	91.56	2.47	101.73	90.71	7.27	100.79
Fenbendazole	LLOQ QC	10	9.87	5.56	98.70	10.15	5.36	101.55
	LQC	30	30.16	3.03	100.53	30.20	4.10	100.66
	MQC	300	301.17	1.87	100.39	297.41	4.00	99.14
	HQC	450	454.03	4.05	100.90	449.30	3.06	99.84
Qxfendazole	LLOQ QC	4	3.93	11.33	98.13	4.18	9.12	104.52
	LQC	12	12.83	3.48	106.94	12.51	5.19	104.29
	MQC	120	123.32	1.08	102.77	119.60	4.67	99.66
	HQC	180	186.56	3.39	103.65	183.95	3.74	102.20

#### 3.2.4 Recovery

The extraction recoveries for the analytes and IS are shown in Table 2. The mean recoveries across three QC levels for PYR ranged from 92.66% to 101.46%, for PRA ranged from 95.07% to 102.55%, for FBT ranged from 93.24% to 102.50%, for FEN ranged from 90.42% to 99.89%, for OXF ranged from 92.58% to 105.38% and for FEN–D3 ranged from 93.37% to 98.04%, respectively. The extraction recoveries for the analytes and IS were all above 90%. The results show that the simplified plasma extraction procedure efficiently extracts all six compounds from dog plasma.

**TABLE 2:** Extraction recovery for the analytes and fenbendazole–D3 (n = 6).

Analyte	Level	Area response		Extraction recovery, (%) (B/A)
		A	B	
Pyrantel	LQC	3,144.322	2,913.510	92.66
	MQC	37,053.699	37,593.218	101.46
	HQC	57,475.880	54,071.148	94.08
Praziquantel	LQC	2,860.737	2,802.062	97.95
	MQC	33,322.353	34,171.646	102.55
	HQC	51,581.389	49,036.528	95.07
Febantel	LQC	1,175.403	1,204.827	102.50
	MQC	15,199.489	15,077.834	99.20
	HQC	23,157.229	21,591.874	93.24
Fenbendazole	LQC	9,731.459	8,798.774	90.42
	MQC	110,194.839	110,071.522	99.89
	HQC	165,414.414	156,937.581	94.88
Qxfendazole	LQC	1,409.799	1,305.230	92.58
	MQC	16,293.469	17,170.556	105.38
	HQC	25,613.659	24,871.638	97.10
Fenbendazole–D3	LQC	7,614.619	7,110.111	93.37
	MQC	8,744.829	8,573.594	98.04
	HQC	8,530.820	8,229.814	96.47

A, mean area response of six replicates prepared by spiking in extracted blank plasma; B, mean area response of six replicates prepared by spiking before extraction.

#### 3.2.5 Matrix effect

The matrix effects for the analytes and IS are presented in Table 3. The mean IS–normalized matrix factors across three QC levels for PYR ranged from 1.022 to 1.073, for PRA ranged from 1.000 to 1.056, for FBT ranged from 0.931 to 1.051, for FEN ranged from 1.007 to 1.108 and for OXF ranged from 1.016 to 1.066, respectively. The variation coefficients of the IS–normalized matrix factors across three QC levels were found to be ≤4.66% for PYR, ≤4.74% for PRA, ≤6.44% for FBT, ≤4.24% for FEN, and ≤5.91% for OXF, respectively. These values were within the acceptance criteria of ≤15%. Hence, These results indicate that the plasma components have negligible effects on the quantification of the analytes under the optimized conditions.

**TABLE 3:** Matrix factors for the analytes and Fenbendazole–D3 (n = 6).

Analyte	Level	Area response		Matrix Factor (A/B)	CV (%)	IS–normalized matrix factor	CV (%)
		A	B				
Pyrantel	LQC	3,144.322	3,049.424	1.031	6.14	1.057	4.66
	MQC	37,053.699	370,68.832	1.000	4.49	1.022	3.00
	HQC	57,475.880	58,067.132	0.990	3.57	1.073	2.79
Praziquantel	LQC	2,860.737	2,823.089	1.013	6.86	1.039	4.74
	MQC	33,322.353	34,087.219	0.978	5.43	1.000	2.36
	HQC	51,581.389	52,899.590	0.975	3.28	1.056	2.23
Febantel	LQC	1,175.403	1,294.659	0.908	7.35	0.931	6.44
	MQC	15,199.489	15,208.414	0.999	7.51	1.021	6.27
	HQC	23,157.229	23,865.053	0.970	4.09	1.051	2.40
Fenbendazole	LQC	9,731.459	9,011.653	1.080	5.17	1.108	4.24
	MQC	110,194.839	111,819.463	0.985	7.79	1.007	3.87
	HQC	165,414.414	174,337.000	0.949	2.64	1.028	1.05
Qxfendazole	LQC	1,409.799	1,382.087	1.020	6.75	1.046	5.91
	MQC	16,293.469	16,393.385	0.994	6.67	1.016	2.88
	HQC	25,613.659	26,033.167	0.984	3.95	1.066	3.53
Fenbendazole–D3	LQC	7,614.619	7,813.497	0.975	3.58	–	–
	MQC	8,744.829	8,941.962	0.978	5.37	–	–
	HQC	8,530.820	9,243.344	0.923	2.96	–	–

A, mean area response of six replicates prepared by spiking in extracted blank plasma; B, Mean area response of six replicates prepared by spiking in mobile phase (neat samples); IS–normalized matrix factor: Analyte matrix factor/IS, matrix factor; –, no data.

#### 3.2.6 Dilution reliability

Spiked dog plasma samples prepared at concentrations above ULOQ for all the analytes were diluted with five folds in five replicates and analyzed. The accuracy results for PYR were between 90.90% and 99.76%, for PRA were between 85.93% and 95.74%, for FBT were between 86.11% and 99.53%, for FEN were between 86.03% and 98.56%, for OXF were 86.90% and 97.50%, respectively, while the precisions (CV) for PYR were 4.44%, for PRA were 4.01%, for FBT were 5.65%, for FEN were 5.18% and for OXF were 4.44%, respectively. These data demonstrate that this dilution method has no impact on the accuracy and precision of sample detection.

#### 3.2.7 Stability experiments

The results of stability experiments in plasma under the mentioned conditions at two QC levels are summarized in Table 4. Samples for short–term stock solution stability at 4°C remained unchanged up to 24 h, respectively, for the analytes and the internal standard (IS). Regarding the long–term stability of the analytes, the stock solution remained stable for 77 days, while that of the IS remained stable for 104 days. The bench–top stability results for the analytes showed that they were stable for at least 8 h at room temperature in plasma samples. Refrigerator stability for the analytes was established up to 30 h. The freeze–thaw stability results indicated that repeated freezing and thawing (three cycles) did not affect the stability of the analytes for samples stored at −80°C. Autosampler stability of the processed samples at 6°C was carried out for 55 h. For short–term stability, plasma samples stored at −20°C were found to be stable for 48 h, while the samples were stable for a minimum period of 120 days.

**TABLE 4:** Stability results for the analytes (n = 5).

Storage conditons	Analyte	Level	Concentration added (ng/mL)	Mean stability sample ±SD (ng/mL)	CV (%)	Accuracy (%)
Bench top stability at room temperature, 8 h	Pyrantel	LQC	12	11.30 ± 0.66	5.81	94.15
		HQC	180	163.22 ± 1.45	0.89	90.68
	Praziquantel	LQC	45	44.28 ± 0.95	2.14	98.41
		HQC	675	645.65 ± 11.69	1.81	95.65
	Febantel	LQC	6	6.10 ± 0.38	6.29	101.59
		HQC	90	88.24 ± 1.25	1.42	98.05
	Fenbendazole	LQC	30	28.75 ± 0.77	2.67	95.83
		HQC	450	412.09 ± 6.95	1.69	91.58
	Qxfendazole	LQC	12	12.08 ± 1.02	8.49	100.65
		HQC	180	162.16 ± 2.99	1.85	90.09
Refrigerator stability at 4°C, 30 h	Pyrantel	LQC	12	11.66 ± 0.93	7.98	97.18
		HQC	180	174.99 ± 6.96	3.98	97.21
	Praziquantel	LQC	45	44.17 ± 3.20	7.25	98.16
		HQC	675	654.44 ± 29.69	4.54	96.95
	Febantel	LQC	6	5.78 ± 0.56	9.69	96.42
		HQC	90	87.67 ± 3.15	3.59	97.41
	Fenbendazole	LQC	30	29.11 ± 1.92	6.61	97.04
		HQC	450	438.32 ± 14.73	3.36	97.40
	Qxfendazole	LQC	12	11.46 ± 0.74	6.48	95.50
		HQC	180	174.31 ± 5.37	3.08	96.84
Freeze–thaw stability after 3rd cycle at −80°C	Pyrantel	LQC	12	11.06 ± 0.55	4.99	92.15
		HQC	180	170.71 ± 4.98	2.92	94.84
	Praziquantel	LQC	45	44.15 ± 2.42	5.49	98.12
		HQC	675	669.52 ± 14.97	2.24	99.19
	Febantel	LQC	6	6.15 ± 0.48	7.84	102.52
		HQC	90	88.31 ± 1.14	1.29	98.12
	Fenbendazole	LQC	30	29.65 ± 1.05	3.53	98.82
		HQC	450	447.72 ± 13.36	2.98	99.49
	Qxfendazole	LQC	12	12.12 ± 0.84	6.90	100.97
		HQC	180	175.01 ± 5.08	2.90	97.23
Autosampler stability at 6°C, 55 h	Pyrantel	LQC	12	12.87 ± 0.28	2.19	107.25
		HQC	180	194.53 ± 3.27	1.68	108.07
	Praziquantel	LQC	45	46.65 ± 1.23	2.63	103.68
		HQC	675	729.27 ± 27.29	3.74	108.04
	Febantel	LQC	6	6.42 ± 0.40	6.24	107.01
		HQC	90	95.98 ± 3.18	3.32	106.65
	Fenbendazole	LQC	30	30.26 ± 0.97	3.19	100.87
		HQC	450	457.91 ± 24.20	5.28	97.40
	Qxfendazole	LQC	12	12.82 ± 0.85	6.62	106.85
		HQC	180	192.72 ± 8.41	4.36	107.07
Short term stability at −20°C, 48 h	Pyrantel	LQC	12	12.05 ± 0.92	7.61	100.41
		HQC	180	193.96 ± 8.82	4.55	107.76
	Praziquantel	LQC	45	45.15 ± 3.68	8.15	100.34
		HQC	675	730.20 ± 19.11	2.62	108.18
	Febantel	LQC	6	5.75 ± 0.51	8.80	96.29
		HQC	90	90.76 ± 4.24	4.67	100.85
	Fenbendazole	LQC	30	30.94 ± 2.18	7.05	103.13
		HQC	450	470.60 ± 13.89	2.95	104.58
	Qxfendazole	LQC	12	11.80 ± 0.79	6.68	98.29
		HQC	180	189.73 ± 8.17	4.30	105.41
Long term stability at −80°C, 120 days	Pyrantel	LQC	12	10.63 ± 0.11	0.99	88.57
		HQC	180	175.05 ± 5.76	3.29	97.25
	Praziquantel	LQC	45	42.50 ± 4.13	9.72	94.44
		HQC	675	615.17 ± 19.69	3.20	91.14
	Febantel	LQC	6	5.86 ± 0.22	3.67	97.70
		HQC	90	89.57 ± 3.12	3.48	99.52
	Fenbendazole	LQC	30	30.69 ± 0.89	2.90	102.31
		HQC	450	455.87 ± 11.17	2.45	101.31
	Qxfendazole	LQC	12	11.28 ± 0.62	5.47	93.96
		HQC	180	175.76 ± 5.23	2.98	97.64

#### 3.2.8 Carry–over

No residues of the analytes and internal standard were detected in the extracted blank sample (without analytes and IS) after subsequent injection of the ULOQ.

### 3.3 Application to a bioequivalence study

The bioequivalence study based on plasma drug concentration represents an efficacious approach to determine the pharmaceutical equivalence or the pharmaceutical substitutability of two products containing the same active substance. As the C_max_ of the parent compound is more sensitive to detecting absorption rate differences among dosage forms than that of the metabolite, the bioequivalence is evaluated based on the concentration of the parent compound. Nevertheless, to comprehensively understand the pharmacokinetic characteristics of the test formulation, the plasma drug concentrations of the metabolites were also determined and their corresponding pharmacokinetic parameters were analyzed. However, the bioequivalence of the metabolites was not calculated. Therefore, in the bioequivalence study of the compound febantel tablets, it is necessary to detect the plasma drug concentrations of five components, namely, PYR, PRA, FBT, FEN and OXF.

The UPLC–MS/MS analytical method was successfully applied to determine the analyte concentrations in dog plasma samples from the bioequivalence study. The study was conducted as an open label, balanced, randomized, crossover, two–treatment, two–period, two–sequence and single–dose design to compare the bioavailability of PYR, PRA, FBT, FEN and OXF between two products in 38 healthy beagle dogs. Each subject received a tablet from the test product (Self–developed preparation) and a tablet from reference product (Drontal^®^ Plus Flavor) under fasting conditions with a washout period of 2 weeks.

**FIGURE 3:**
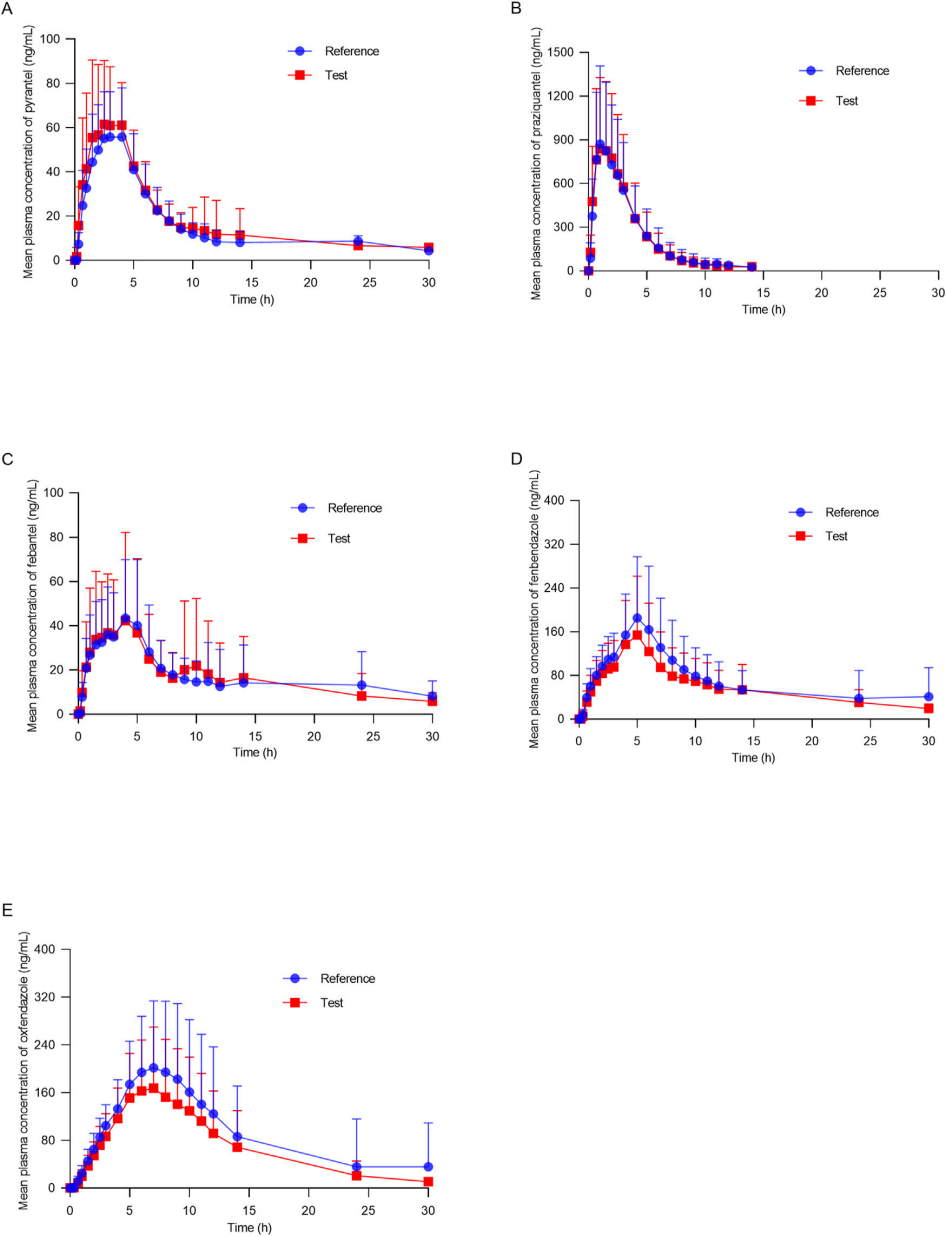
Mean plasma concentration–time profiles of **(A)** pyrantel, **(B)** praziquantel, **(C)** febantel, **(D)** fenbendazole and **(E)** oxfendazole after oral of compound febantel formulations (test and reference) to 38 healthy beagle dogs.

Figure 3 shows the mean plasma concentration versus time profiles of the anlytes in 38 healthy dogs. The mean pharmacokinetic parameters obtained from both formulations are represented in Table 5. The results demonstrated the pharmacokinetic characteristics after oral administration of the reference tablets and the test tablets of compound febantel in dogs. Compared with the former study (Klausz et al., 2015), this one offered more comprehensive pharmacokinetic parameters and a clearer insight into the absorption and metabolism of compound febantel tablets, providing a reference for clinical use. The mean C_max_ values obtained for PYR in the present work were 80.49 ng/mL for test products and 69.17 ng/mL for reference products. These parameters were comparable with a similar bioequivalence study involving 20 healthy beagle dogs in which C_max_ values obtained for PYR were 78.5 ng/mL for test products and 81.1 ng/mL for reference products. However, the C_max_ values for the other four analytes were higher compared to those in 20 beagle dogs given the identical dose strength, especially for PRA and FBT. Other parameters could not be compared because the data was not provided (Klausz et al., 2015). It is observable from these data that a significant inter–individual variability existed for every compound under test. This difference could be attributed to several factors including type of food, gender, age, weight and other renal and hepatic functions. However, the intra–individual variabilities, which refer to the measured concentrations after the test formulation and the reference formulation were administered to the same animal, were much lower in all cases. Therefore, the geometric mean ratios of the C_max_, AUC_0–30_, AUC_0–inf_ and their 90% confidence intervals were between 82.07% and 122.74%, which are within the defined bioequivalence range of 80%–125% (Table 6). The results validated the equivalence of the two products in terms of rate and extent of absorption.

**TABLE 5:** Mean Pharmacokinetic parameters (±SD) after oral administration of compound febantel tablets in 38 healthy beagle dogs under fasting condition.

Parameters	Products	Pyrantel	Praziquantel	Febantel	Fenbendazole	Qxfendazole
C_max_ (ng/mL)	T	80.49 ± 33.61	929.39 ± 499.02	63.26 ± 45.97	181.95 ± 96.08	183.58 ± 95.73
	R	69.17 ± 21.12	957.04 ± 537.42	58.66 ± 28.55	212.35 ± 104.41	224.25 ± 111.75
AUC_0–30_ (h. ng/mL)	T	422.63 ± 139.70	3,315.20 ± 1967.65	436.23 ± 345.62	1,441.91 ± 742.81	1,789.33 ± 1,001.10
	R	378.04 ± 102.58	3,267.67 ± 1827.15	427.68 ± 279.07	1,689.33 ± 881.68	2,099.86 ± 1,132.52
AUC_0–inf_ (h ng/mL)	T	488.59 ± 185.74	3,365.39 ± 1977.29	514.04 ± 361.93	1,682.54 ± 859.09	1,908.34 ± 1,070.93
	R	429.16 ± 117.30	3,317.89 ± 1838.60	526.57 ± 471.25	1,893.90 ± 934.66	2,550.16 ± 1,693.95
T_max_ (h)	T	2.87 ± 1.06	1.48 ± 0.67	4.59 ± 2.86	5.20 ± 3.10	7.08 ± 2.65
	R	3.08 ± 0.98	1.45 ± 1.05	4.46 ± 2.86	5.00 ± 3.56	6.97 ± 3.47
t_1/2_ (h)	T	4.27 ± 2.03	1.66 ± 0.41	7.59 ± 9.01	6.96 ± 8.33	3.83 ± 3.03
	R	4.87 ± 4.38	1.65 ± 0.46	8.64 ± 11.31	5.76 ± 3.70	4.17 ± 2.45
K_el_ (1/h)	T	0.19 ± 0.07	0.44 ± 0.12	0.14 ± 0.08	0.16 ± 0.07	0.24 ± 0.10
	R	0.20 ± 0.08	0.45 ± 0.12	0.16 ± 0.09	0.15 ± 0.07	0.22 ± 0.10

C_max_, the maximum plasma concentration; AUC_0–30_, the area under the plasma concentration–time curve from time zero to the last sampling time; AUC_0–inf_, the area under the plasma concentration–time curve from time zero to infinity; T_max_, the time to reach C_max_; t_1/2_, elimination half–life; K_el_, elimination rate constant; T, test; R, reference.

**TABLE 6:** Comparison of treatment ratios and 90% CIs of natural log (Ln)–transformed parameters of the analytes in the bioequivalence study.

Analytes	Parameters	GMR (Test/Reference, %)	90% CI (Lower–Upper)
Pyrantel	C_max_ (ng/mL)	112.68	103.45–122.74
	AUC_0–30_ (h. ng/mL)	110.18	102.52–118.42
	AUC_0–inf_ (h. ng/mL)	111.84	102.53–121.99
Praziquantel	C_max_ (ng/mL)	97.63	89.58–106.40
	AUC_0–30_ (h. ng/mL)	101.83	93.38–111.04
	AUC_0–inf_ (h. ng/mL)	101.79	93.44–110.89
Febantel	C_max_ (ng/mL)	98.40	84.59–114.47
	AUC_0–30_ (h. ng/mL)	96.37	82.07–113.16
	AUC_0–inf_ (h. ng/mL)	98.04	82.70–116.22

GMR, geometric mean ratio; CI, confidence interval.

## 4 Conclusion

A selective, sensitive, simple and rapid UPLC–MS/MS method was developed and fully validated for the quantification of PYR, PRA, FBT, FEN and OXF in dog plasma. The method presents obvious advantages over the existing analytical method in terms of smaller plasma volume for processing, better sensitivity, higher selective, faster analysis time and simpler sample handing procedure (Table 7). The developed assay method was successfully applied to a bioequivalence study in dogs. The outcomes of the bioequivalence test conclusively indicated that the test formulation exhibited bioequivalence with the reference formulation. Consequently, the test formulation is capable of substituting the original research formulation, thereby presenting an supplementary alternative for clinical utilization.

**TABLE 7:** Method comparison.

Comparison	Method in this article	Existing method (Klausz et al., 2015)
Instrument	UPLC–MS/MS	HPLC–MS
Plasma volume for processing	100 μL	500 μL
Sample handing procedure	Simple protein precipitation combined with liquid–liquid extraction	Complex solid–phase extraction
Analysis time	9 min	23 min
Sensitivity	LLOQ of febantel: 2 ng/mL	LLOQ of febantel: 3 ng/mL

UPLC–MS/MS, ultra–performance liquid chromatography–tandem mass spectrometry; HPLC–MS, high–performance Liquid chromatography–mass spectrometry; LLOQ, lower limit of quantification.

## Data Availability

The original contributions presented in the study are included in the article/supplementary material, further inquiries can be directed to the corresponding author.
